# RAB4A DRIVES PROINFLAMMATORY CD4^+^ T CELL SIGNALING VIA CD38-DEPENDENT NAD^+^ METABOLISM

**DOI:** 10.21203/rs.3.rs-6787101/v1

**Published:** 2025-06-12

**Authors:** Joy S. Park, Daniel Krakko, Jessica Nolan, Brandon Wyman, Mahsa Sadeghzadeh, Andras Perl

**Affiliations:** 1 Departments of Medicine, State University of New York, Upstate Medical University, Norton College of Medicine, Syracuse, New York 13210;; 2 Biochemistry and Molecular Biology, State University of New York, Upstate Medical University, Norton College of Medicine, Syracuse, New York 13210;; 3 Microbiology and Immunology, State University of New York, Upstate Medical University, Norton College of Medicine, Syracuse, New York 13210;

**Keywords:** CD4^+^ T cells, Rab4A, CD38, IL-2, endosome traffic, NAD^+^, metabolism, mTOR, autoimmunity, systemic lupus erythematosus

## Abstract

Rab4A, a small GTPase overexpressed in T cells of patients with systemic lupus erythematosus (SLE), has been shown to activate mechanistic target of rapamycin (mTOR) signaling, which promotes proinflammatory T cell development and predisposes to nephritis in SLE. In this study, we demonstrate that Rab4A facilitates the endocytic recycling and surface expression of CD38, which, in turn, triggers NAD^+^ depletion, activates mTOR complex 1, and suppresses interleukin-2 (IL-2) production in CD4^+^ T cells. Rab4A-driven CD38-mediated NAD^+^ depletion elicits the accumulation of nicotinamide and ADP-ribose and secondary depletion of cyclic ADP-ribose. Surprisingly, rapamycin further enhanced CD38 expression and reduced IL-2 secretion, suggesting that IL-2 depletion is mTOR-independent. Alternatively, Rab4A-driven upregulation of CD38 promoted STAT3 expression and its acetylation, as well as FOXO1 expression, which underlies IL-2 depletion in CD4^+^ T cells. These findings reveal a novel Rab4A-driven CD38 signaling axis that links receptor trafficking to proinflammatory metabolic pathways, providing new targets for treatment in SLE.

## INTRODUCTION

Systemic lupus erythematosus (SLE) is a chronic autoimmune disease characterized by widespread inflammation and immune dysregulation, affecting 20–150 people per 100,000 globally^1,2^. A key feature of SLE pathogenesis is the imbalance between proinflammatory and anti-inflammatory T cells^3^. In SLE patients, proinflammatory interleukin (IL)-17-expressing T helper 17 (T_H_17) cells are expanded^4^, while regulatory T cells (T_reg_), which maintain immune tolerance, are reduced^5^. IL-2, a cytokine critical for T_reg_ differentiation^6^ and suppressing T_H_17 expansion^7^, is notably diminished in SLE^8^, contributing to disease pathogenesis. However, the mechanisms underlying reduced IL-2 production remain incompletely understood. Human T lymphotropic virus type I-related endogenous retroviral sequence (HRES-1) encoded HRES-1/Rab4 (Rab4A), a small GTPase that regulates recycling of early endosomes, has been identified as a key driver of SLE pathogenesis^3^. Rab4A is overexpressed in SLE patient T cells^3,9^ and activates mTOR^10^, a pathway that promotes inflammatory T cells development in SLE and other autoimmune diseases^11^. Moreover, Rab4A regulates the recycling of endocytosed receptors, including CD4 and CD3ζ^3,9^, thereby influencing immune cell function^12^.

CD38, an NAD^+^_-_hydrolyzing ectoenzyme and marker of immune cell activation^13^, is upregulated in SLE T cells compared to healthy controls, and its expression correlates with disease activity^14,15^. Notably, mouse CD4^+^ T cells polarized into T_H_17 cells exhibit higher CD38 expression than other CD4^+^ T cell subsets, such as T_H_1 cells^16^, and recent studies have shown that CD38 enhances the phosphorylation of AKT at Thr308^17^, a modification essential for mTORC1 activation^18^. Beyond hydrolyzing and depleting NAD^+^, CD38 generates adenosine diphosphate ribose (ADPR) and nicotinamide, exerting influence on proinflammatory signaling^19,20^. In this study, we unveiled the dominant role of Rab4A in CD38 recycling and expression, as well as its impact on mTOR activation and IL-2 production in CD4^+^ T cells.

## RESULTS

### Rab4A promotes CD38 expression

**Figure 1 |:**
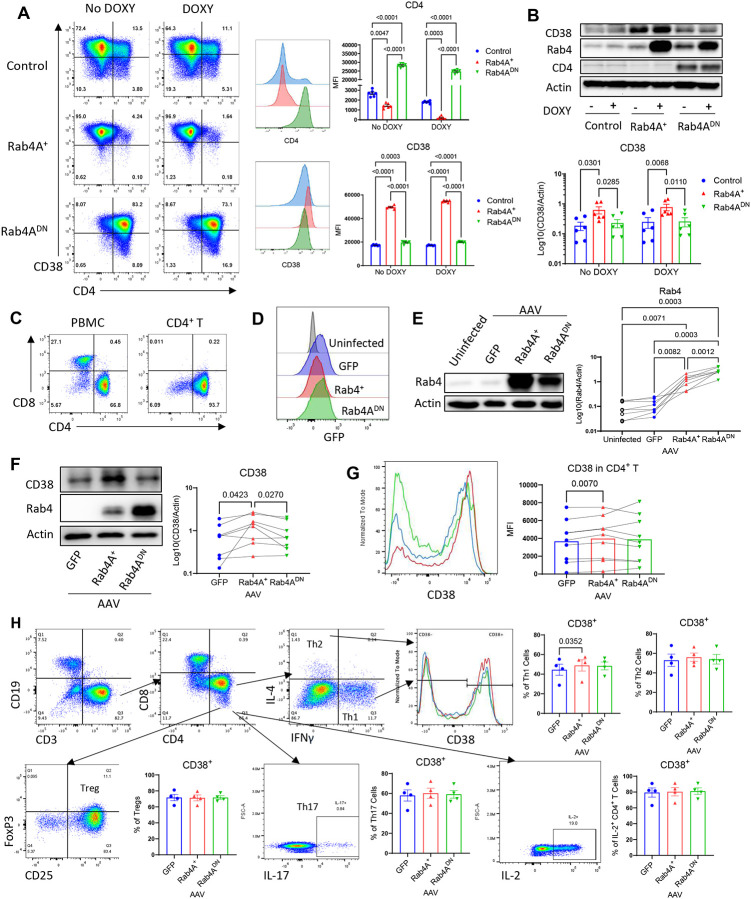
Rab4A promotes CD38 expression and cell surface recycling in Jurkat and primary human CD4^+^ T cells. **(A)** Flow cytometry (n = 6 independent replicates) and **(B)** Western blot analysis (n = 6 independent replicates, 2 separate experiments) show that Rab4A-overexpressing (Rab4A^+^) Jurkat cells exhibit increased CD38 expression compared to control and dominant-negative Rab4A (Rab4A^DN^) Jurkat cells. CD38 expression is significantly upregulated in Rab4A^+^ cells. CD4 expression is included for confirmation. **(C)** CD4^+^ T cells were isolated from healthy donor PBMCs (n = 8), confirmed by flow cytometry. **(D)** Flow cytometry and **(E)** Western blot confirm AAV-mediated transduction of Rab4A^+^ and the dominant-negative mutant, Rab4A^DN^, in primary human CD4^+^ T cells. **(F)** Western blot and **(G)** flow cytometry demonstrate increased CD38 expression in Rab4A^+^ CD4^+^ T cells compared to GFP vector controls. **(H)** Rab4A^+^ T_H_1 cells (CD3^+^ CD4^+^ CD8^−^ IL-4^−^ IFNγ^+^) (n = 4 healthy donors) exhibit a significantly higher percentage of CD38^+^ cells than GFP vector controls; no significant difference in CD38 expression was observed in T_H_2, T_reg_, T_H_17, and IL-2^+^ subsets. Statistical significance was determined by **(A)** two-way ANOVA with Sidak correction for multiple comparisons; **(B)** paired two-way ANOVA with Tukey’s correction for multiple comparisons; and (**E-H)** paired one-way ANOVA with Tukey’s correction for **(E, G)** and Dunnett’s correction for **(F, H)** for multiple comparisons. DOXY = doxycycline.

To investigate the effects of Rab4A, we utilized the Jurkat human CD4^+^ T cell line. Rab4A has previously been shown to regulate receptor recycling, either returning them to the cell surface, as documented for CD71 and CD98^21^, or targeting them for lysosomal degradation, as demonstrated for CD4^3,22^. Metabolic stress also involves NAD^+^ depletion, which has been attributed to overexpression of CD38 in SLE patients^23,24^. Since CD38 is internalized via endosomes^25^, we examined whether CD38 expression is controlled via recycling by Rab4A. In accordance with earlier findings, CD4 levels, both total protein levels and surface expression, were reduced in Jurkat cells overexpressing Rab4A (Rab4A^+^), while CD4 expression was markedly enhanced in Jurkat cells overexpressing dominant-negative Rab4A (Rab4A^DN^; Figure 1A). Flow cytometry revealed that Rab4A^+^ Jurkat cells exhibited significantly increased CD38 surface expression as compared to Jurkat cells transduced with control vector (Control) and Rab4A^DN^ vector (Figure 1A). Western blot analysis confirmed elevated total CD38 protein levels in Rab4A^+^ cells relative to both control and Rab4A^DN^ Jurkat cells (Figure 1B).

To validate these findings in primary human T cells, we isolated CD4^+^ T cells from peripheral blood mononuclear cells (PBMCs) of healthy donors and transduced adeno-associated virus (AAV) bi-cistronic expression vectors producing GFP and wild-type Rab4A (Rab4A^+^), dominant-negative mutant Rab4A^S27N^ (Rab4A^DN^), and GFP alone (GFP). Flow cytometry confirmed successful CD4^+^ T cell isolation (Figure 1C), and GFP expression indicated efficient AAV-mediated Rab4A transduction (Figure 1D). Western blot analysis verified Rab4A overexpression in these primary CD4^+^ T cells (Figure 1E). Similar to Jurkat cells, Rab4A^+^ CD4^+^ T cells exhibited significantly increased CD38 expression, as indicated by both Western blot (Figure 1F) and flow cytometry (Figure 1G). Moreover, Rab4A^+^ moderately expanded interferon (IFN)γ-producing CD38^+^ T helper type 1 (T_H_1) cells (Figure 1H).

To assess the impact of Rab4A on lysosomal trafficking in primary human CD4^+^ T cells, we evaluated the colocalization of CD4 with LysoTracker Red (LTR) by confocal microscopy. Rab4A^+^ CD4^+^ T cells showed increased CD4-LTR colocalization compared to the GFP vector control and Rab4A^DN^ cells (**Supplementary Figure S1**), which was consistent with targeting of CD4 by Rab4 to the lysosome^26^.

**Figure 2 |:**
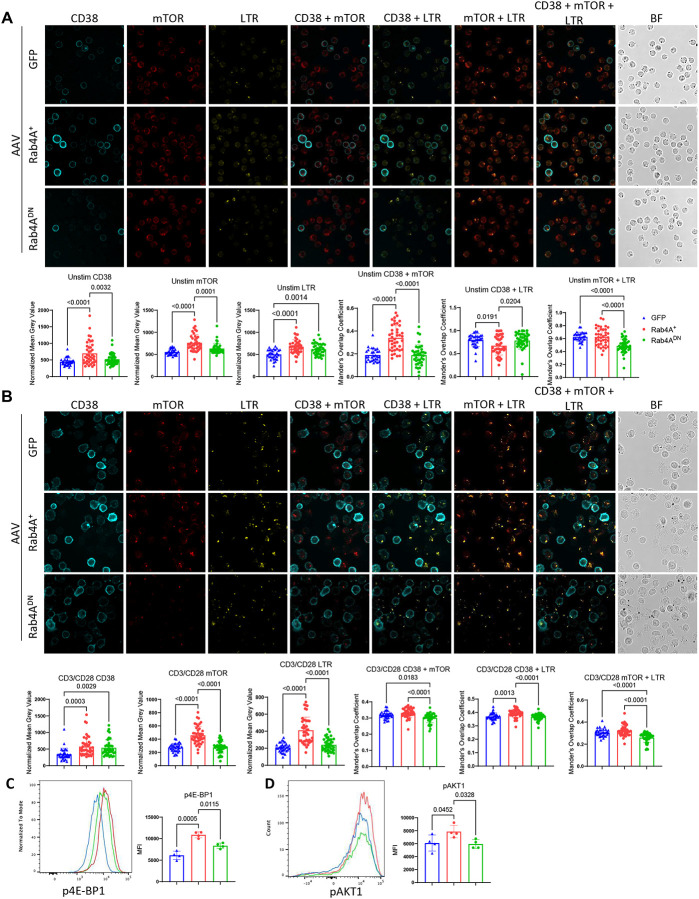
Rab4A promotes CD38 and mTOR expression in human primary CD4^+^ T cells. In Rab4A variant AAV-infected CD4^+^ T cells from healthy donors (n = 5), **(A)** confocal microscopy shows increased expression of CD38 and mTOR in Rab4A^+^ CD4^+^ T cells compared to GFP vector control and Rab4A^DN^ cells. Rab4A^+^ cells display enhanced colocalization of CD38 and mTOR, but reduced colocalization of CD38 with LysoTracker Red (LTR). Rab4A^DN^ cells exhibit reduced colocalization of mTOR and LTR compared to GFP vector control and Rab4A^+^ cells. **(B)** CD3/CD28 co-stimulation further increases CD38 expression in Rab4A^+^ and Rab4A^DN^ cells relative to control. Rab4A^+^ cells show increased mTOR and LTR expression, along with enhanced CD38/LTR colocalization, while Rab4A^DN^ cells exhibit reduced CD38/mTOR and mTOR/LTR colocalization. By flow cytometry, Rab4A^+^ CD4^+^ T cells (n = 4 healthy donors) display elevated phosphorylation of **(C)** mTORC1 substrate, 4E-BP1 (p4E-BP1), and **(D)** mTORC2 substrate, AKT1 (pAKT1), relative to controls. Statistical significance was determined by one-way ANOVA with Tukey’s correction for **(A, B)** and Dunnett’s correction for **(C, D**) for multiple comparisons. Error bars represent mean ± SEM.

Confocal microscopy revealed elevated CD38 expression in Rab4A^+^ cells compared to GFP control and Rab4A^DN^ cells (Figure 2A). Rab4A overall promoted the accumulation of CD38, mTOR, and LTR^+^ lysosomes in Rab4A^+^ primary human CD4^+^ T cells (Figure 2A). Notably, colocalization of CD38 with LTR was reduced, suggesting that Rab4A enhanced CD38 recycling back to the cell surface rather than targeting it for lysosomal degradation (Figure 2A). Upon CD3/CD28 co-stimulation, given that CD38 is known as a T cell activation marker^27^, Rab4A^+^ and Rab4A^DN^ CD4^+^ T cells displayed further modulation of CD38 expression. CD38 levels remained elevated in Rab4A^+^ cells compared to GFP vector controls. Rab4A^DN^ cells also exhibited elevated CD38 expression upon stimulation, which was attributed to lesser colocalization with LTR in comparison to that noted in Rab4A^+^ CD4^+^ T cells (Figure 2B).

Recycling of CD38 was investigated in lupus-prone mice carrying constitutively active Rab4A^Q72L^ alleles (B6.TC/Rab4A^Q72L^) and mice lacking Rab4A in T cells (B6.TC/Rab4A^Q72L^_-_KO)^21^. As shown in **Supplementary Figure S2**, recycling and surface expression of CD38 were enhanced by Rab4A in CD4^+^ T cells, CD8^+^ T cells, and CD19^+^ B cells, but not in double-negative (DN) T cells or non-B/non-T cells of B6.TC/Rab4A^Q72L^ mice relative to B6.TC/Rab4A^Q72L^_-_KO controls. Rab4A also promoted the partitioning of CD38 into the plasma membrane in Jurkat cells (**Supplementary Figure S3**). Together, these findings demonstrate that Rab4A regulates CD38 expression in both Jurkat and primary CD4^+^ T cells. By promoting CD38 recycling and surface expression, Rab4A may enhance downstream signaling. Given that Rab4A activates mTOR^10^ and CD38 has been previously associated with mTOR signaling^17^, we next investigated whether Rab4A-mediated CD38 expression contributes to mTOR activation.

### Rab4A triggers CD38-dependent mTOR activation

CD38 has been implicated in metabolic regulation of T cell signal transduction through its role as a NAD^+^ hydrolase^20^, and recent studies suggest that CD38 can modulate mTOR signaling^17^. Having established that Rab4A enhances CD38 recycling and surface expression, we next examined whether this upregulation contributes to mTOR activation. As shown by confocal microscopy, Rab4A promoted the colocalization of CD38 with mTOR (Figure 2A), which was reduced in Rab4A^DN^ CD4^+^ T cells relative to Rab4A^+^ cells (Figures 2A and 2B). Rab4A also promoted the co-localization of mTOR with LTR^+^ lysosomes with and without TCR stimulation (Figures 2A and 2B). In contrast, Rab4A^DN^ cells showed reduced mTOR-LTR colocalization (Figure 2B). Given that mTORC1 activation requires its translocation to the lysosome^28,29^, these observations support the notion that Rab4A enhances mTOR activation through traffic to lysosomes^21^.

**Figure 3 |:**
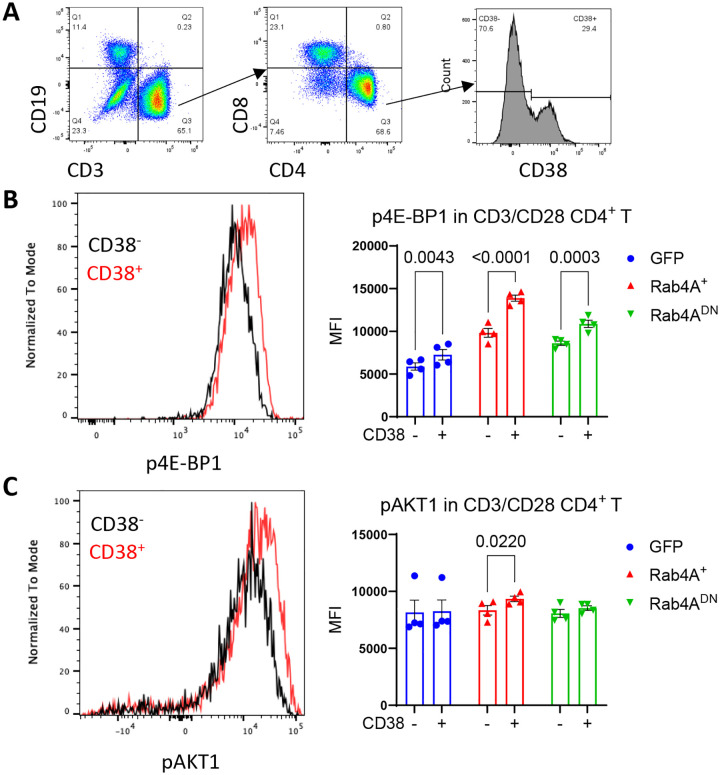
Rab4A-associated mTOR activation correlates with CD38 in CD4^+^ T cells. **(A)** Gating strategy of gating on CD38^−^ and CD38^+^ CD4^+^ T cells. In GFP, Rab4A^+^, and Rab4A^DN^ vector-transduced primary human CD4^+^ T cells from healthy donors (n = 4), **(B)** p4E-BP1 is elevated in CD38^+^ compared to CD38^−^ cells across all conditions, whereas **(C)** pAKT1 is selectively increased in CD38^+^ cells only in Rab4A^+^ CD4^+^ T cells. Statistical analysis was performed using an unpaired t-test. Data are presented as mean ± SEM.

**Figure 4 |:**
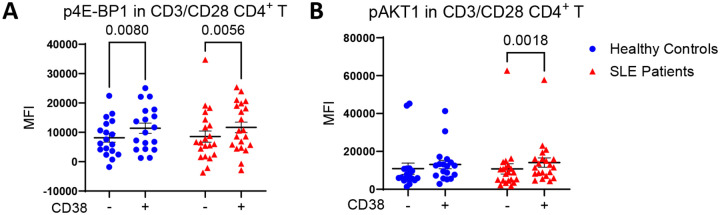
mTOR activation is increased in CD38^+^ CD4^+^ T cells of SLE patients. **(A)** In human primary CD4^+^ T cells, flow cytometry shows that CD38^+^ cells exhibit increased phosphorylation of the mTORC1 substrate 4E-BP1 in both SLE patients (n = 23) and age-, race-, and sex-matched healthy donors (n = 23) compared to CD38^−^ cells. **(B)** CD38^+^ cells also show increased phosphorylation of the mTORC2 substrate AKT1, but only in SLE patients. Statistical analysis was performed using paired two-way ANOVA with Sidak correction for multiple comparisons. Data are presented as mean ± SEM.

To assess mTOR function, we analyzed phosphorylation of mTORC1 and mTORC2 substrates, 4E-BP1 and AKT1, respectively. Flow cytometry revealed that Rab4A^+^ increased the phosphorylation of both 4E-BP1 (Figure 2C) and AKT1 relative to GFP vector and Rab4A^DN^ controls in CD4^+^ T cells (Figure 2D). Next, we compared the extent of Rab4A-induced mTOR activation between CD38^+^ and CD38^−^ primary CD4^+^ T cells (Figure 3A). While CD38 conferred increased activation of mTORC1 independent of Rab4A (Figure 3B), it preferentially enhanced mTORC2 in Rab4A^+^ cells overexpressing Rab4A (Figure 3C). Supporting these findings in the setting of SLE, PBMCs from SLE patients and age-, sex-, and ethnicity-matched healthy donors showed that CD38^+^ CD4^+^ T cells had significantly higher p4E-BP1 levels as compared to CD38^−^ CD4^+^ T cells (Figure 4A). Notably, pAKT1 was solely elevated in CD38^+^ CD4^+^ T cells in SLE patients (Figure 4B) and exhibited selective responsiveness to Rab4A overexpression (Figure 3C).

To further evaluate whether this mTOR activation is CD38-dependent, we generated CRISPR-mediated CD38 deletion (CD38^KO^) in Rab4A-modified Jurkat cells (**Supplementary Figures S4A and S4B**). Both Western blot and flow cytometry analyses showed that CD38 deletion in Rab4A^+^ CD38^KO^ cells blocked the phosphorylation of 4E-BP1 and AKT1 as compared to Rab4A^+^ CD38^WT^ controls (**Supplementary Figures S4C-S4F**). Collectively, these findings indicate that Rab4A promotes mTOR activation via CD38 by enhancing its expression and co-localization with mTOR, establishing CD38 as a regulatory checkpoint between Rab4A-driven mTOR activation in CD4^+^ T cells.

### Rab4A regulates IL-2 production via CD38

In addition to regulating mTOR, CD38 has been implicated in modulating the expression of IL-2, a key cytokine for immune homeostasis and tolerance^30^. IL-2 promotes T_reg_ differentiation^6^ and suppresses proinflammatory T_H_17 cell^7^ expansion, and its production is reduced in SLE T cells^8^. Since Rab4A regulates the trafficking of receptors critical for T cell receptor (TCR) signaling, including CD3ζ and CD4^3,9^, and TCR engagement is critical for IL-2 transcription^31^, we hypothesized that Rab4A may serve as an upstream modulator of IL-2 production. Therefore, we investigated whether Rab4A, through its regulation of CD38, also influences IL-2 production.

**Figure 5 |:**
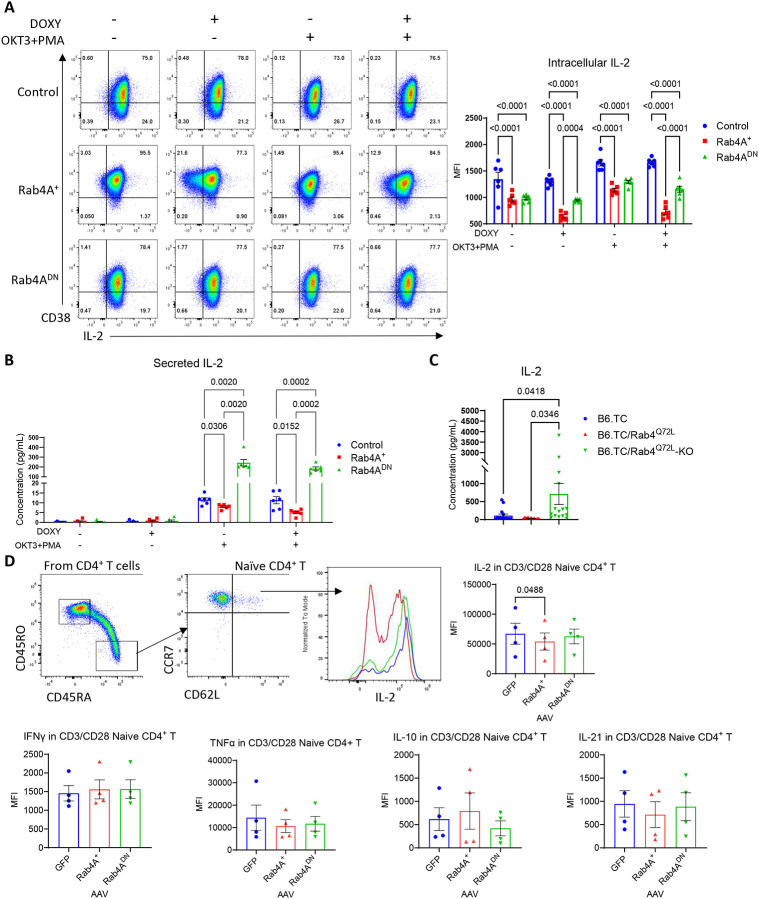
Rab4A reduces IL-2 expression and secretion in Jurkat cells, B6 triple congenic (TC) mice, and primary human naïve CD4^+^ T cells. **(A)** Flow cytometry confirms the upregulation of CD38 in Rab4A^+^ Jurkat cells compared to control and Rab4A^DN^ cells. Intracellular IL-2 production (n = 5 independent cell culture replicates) and **(B)** IL-2 secretion (n = 6 independent cell culture replicates) are significantly reduced in Rab4A^+^ cells, while Rab4A^DN^ cells exhibit significantly increased IL-2 secretion. **(C)** Serum cytokine profiling of B6.TC mice with T cell-specific Rab4A knockout (Rab4A^Q72L^_-_KO) reveals elevated cytokine levels, including IL-2, compared to Rab4A constitutively active mutant (Rab4A^Q72L^) mice. The numbers of mice in each genotype were as follows: B6.TC (n = 15), B6.TC/Rab4A^Q72L^ (n = 11), and B6.TC/Rab4A^Q72L^_-_KO (n = 15). **(D)** In primary human naïve CD4^+^ T cells (CD4^+^CD45RA^+^CD45RO^−^CD62L^+^CCR7^+^) from healthy donors (n = 4), Rab4A^+^ transduction reduces intracellular IL-2 levels compared to controls. IFNγ, TNFα, IL-10, and IL-21 are unchanged with Rab4A AAV transduction. Statistical analyses include two-way ANOVA with Sidak’s correction for multiple comparisons for **(A-C)** and paired one-way ANOVA with Dunnett’s correction for multiple comparisons for **(D)**. Data are presented as mean ± SEM.

Flow cytometry showed that Rab4A reduced intracellular IL-2 production in Rab4A^+^ Jurkat cells as compared to control cells transduced with vector alone or Rab4A^DN^ Jurkat cells (Figure 5A). Moreover, cytometric bead array (CBA) analysis confirmed that upon anti-CD3 (OKT3) and phorbol 12-myristate 13-acetate (PMA) stimulation, Rab4A^+^ cells secreted significantly less IL-2 into the cell culture supernatant as compared to vector control cells, while Rab4A^DN^ cells showed markedly increased IL-2 secretion (Figure 5B).

To examine the potential involvement of Rab4A in IL-2 depletion in SLE, we analyzed sera of lupus-prone B6.TC mice. Importantly, T cell-specific Rab4A deletion markedly increased IL-2 secretion in B6.TC/Rab4A^Q72L^_-_KO mice relative to B6.TC and B6.TC/Rab4A^Q72L^ controls (Figure 5C). Moreover, human naïve CD4^+^ T cells (CD4^+^ CD45RA^+^ CD45RO^−^ CD62L^+^ CCR7^+^) with AAV-mediated overexpression of Rab4A (Rab4A^+^) also exhibited reduced intracellular IL-2 production compared to controls (Figure 4D). Expressions of IFNγ, tumor necrosis factor (TNF)α, IL-4, and IL-10 were not affected by Rab4A (Figure 4D).

**Figure 6 |:**
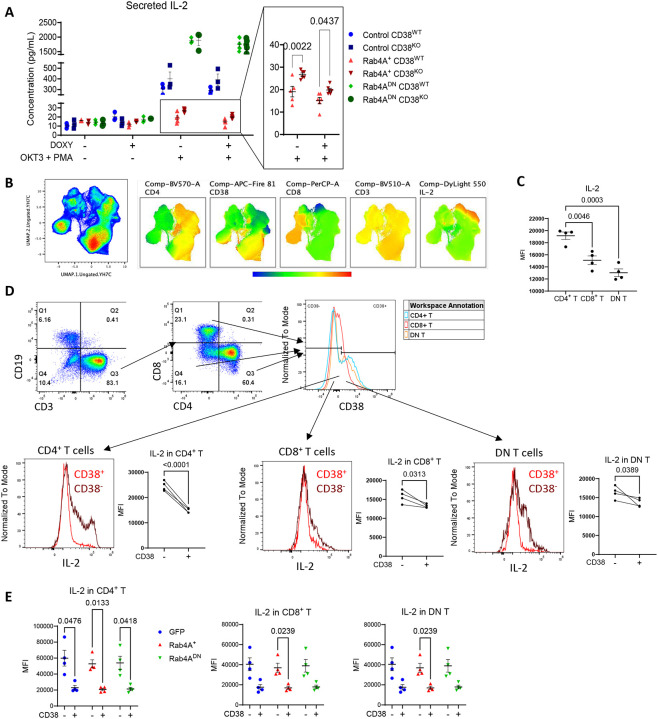
CD38 suppresses IL-2 production in Jurkat cells and primary human CD4^+^ T, CD8^+^ T, and DN T cells from healthy donors. **(A)** In Jurkat cells (n = 3–6 independent cell culture replicates, depending on cell line and stimulation conditions), following OKT3 + PMA stimulation, IL-2 secretion is significantly increased in the Rab4A^+^ CD38^KO^ cells compared to the Rab4A^+^ CD38^WT^ control cells. **(B)** UMAP analysis of primary T cells from healthy donors (n = 4) reveals an inverse relationship between CD38 and IL-2 expression. **(C)** CD4^+^ T cells produce more IL-2 than CD8^+^ T and DN T cells. **(D)** Intracellular IL-2 levels are significantly higher in CD38^−^ compared to CD38^+^ CD4^+^ T, CD8^+^ T, and DN T cells. **(E)** In Rab4A AAV-transduced CD4^+^, CD8^+^, and DN T cells, intracellular IL-2 levels were significantly higher in CD38^−^ compared to CD38^+^ cells, especially in the Rab4A^+^ cells. Statistical significance was determined using a paired 2-tailed t-test for **(C-D**) and two-way ANOVA with Sidak’s corrections for multiple comparisons for **(A, E)**. Data are presented as mean ± SEM.

Given CD38’s role in immune regulation^32^, we next tested whether Rab4A suppresses IL-2 via CD38. CRISPR-mediated deletion of CD38 in CD38^KO^ in Rab4A^+^ Jurkat cells restored IL-2 production, indicating CD38’s involvement (Figure 6A). In primary human PBMCs from healthy donors (n = 4), IL-2 levels were inversely correlated with CD38 expression (Figure 6B). While IL-2 production was generally higher in CD4^+^ than CD8^+^ T cells and DN T cells (Figure 6C), CD38^−^ cells within each T cell subset produced significantly more IL-2 than their CD38^+^ counterparts (Figure 6D). In AAV-transduced primary human T cells overexpressing Rab4A, CD38^−^ cells consistently exhibited significantly higher intracellular IL-2 levels than CD38^+^ cells across all subsets—CD4^+^, CD8^+^, DN T cells—with the most consistent and pronounced effect observed in Rab4A^+^ cells (Figure 6E).

We also assessed co-expression of IL-2 with other T_H_1-associated cytokines in Rab4A-AAV-infected PBMCs (Figure 1H). Across CD4^+^, CD8^+^, and DN T cells, CD382210032 cells consistently showed higher frequencies of TNFα^+^ IL-2^+^, IFNγ^+^ IL-2^+^, and TNFα^+^ IFNγ^+^ cells than CD38^+^ cells, mostly independent of Rab4A expression (**Supplementary Figure S5**). These findings demonstrate that Rab4A suppresses IL-2 production in T cells via a CD38-dependent pathway across multiple T cell subsets, suggesting a broader immunomodulatory role for the Rab4A-CD38 axis across multifunctional T_H_1 cells.

### Rab4A-driven CD38-mediated IL-2 depletion is independent of mTOR

mTOR signaling plays a pivotal role in T cell activation and differentiation, and prior studies have shown that mTOR activity can influence IL-2 production in dendritic cells^33^. Since Rab4A enhances CD38 expression and promotes mTOR activation, we next investigated whether Rab4A-mediated suppression of IL-2 is mTOR-dependent. To address this, we treated control, Rab4A^+^, and Rab4A^DN^ Jurkat cells with rapamycin, an mTOR inhibitor, and assessed both CD38 expression and IL-2 production. Western blot probed for phosphorylation of mTORC1 substrates, 4E-BP1 and S6K, and mTORC2 substrate, AKT1, confirmed successful inhibition of mTOR activity (**Supplementary Figure S6A**). Flow cytometry revealed that rapamycin treatment significantly increased CD38 expression in Rab4A^+^ cells while intracellular IL-2 levels decreased across all cell types (**Supplementary Figure S6B**). These results indicate that although Rab4A enhances mTOR activation via CD38, Rab4A-mediated IL-2 depletion is CD38-dependent but mTOR-independent. This uncoupling implies that CD38 regulates IL-2 production via alternative signaling pathways distinct from its role in mTOR activation.

### Rab4A promotes NAD^+^ depletion via CD38

**Figure 7 |:**
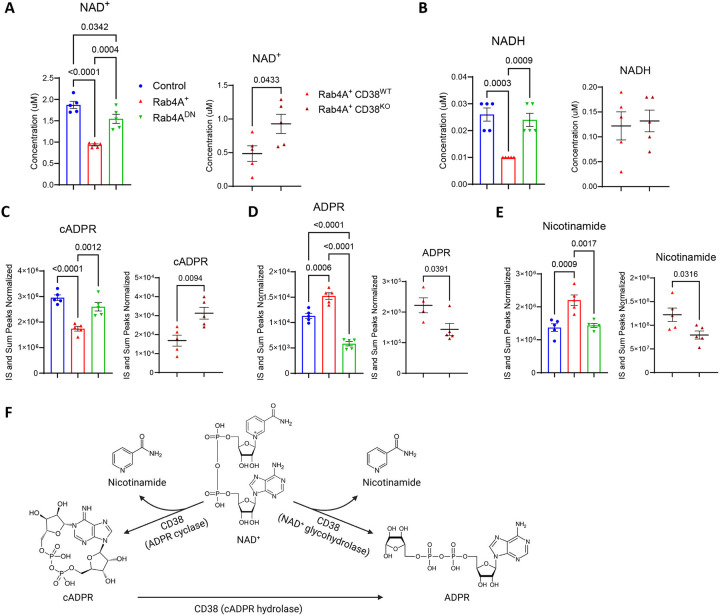
Rab4A promotes NAD^+^ metabolism through CD38 in Jurkat cells. **(A-D)** Targeted LC-MS/MS analysis of Jurkat cells (n = 5 independent cell culture replicates) reveals that **(A)** intracellular NAD^+^ levels are significantly reduced in Rab4A^+^ Jurkat cells, where CD38 expression is elevated, compared to control and Rab4A^DN^ cells. CD38^KO^ in Rab4A^+^ cells restores NAD^+^ levels. **(B)** NADH levels are also reduced in Rab4A^+^ cells compared to controls but remain unchanged following CD38^KO^. **(C-E)** Untargeted LC-MS/MS analysis shows that **(D-E)** ADPR and nicotinamide levels are significantly increased in Rab4A^+^ cells, and CD38^KO^ in Rab4A^+^ cells restores both levels. **(C)** cADPR levels are reduced in Rab4A^+^ cells, and CD38^KO^ in Rab4A^+^ cells restores cADPR levels. **(F)** CD38 is a multifunctional ectoenzyme with NAD^+^ glycohydrolase activity, converting NAD^+^ into ADP-ribose (ADPR) and nicotinamide, as well as ADP-ribosyl cyclase and cADPR hydrolase activities. Statistical significance was determined by two-way ANOVA with Sidak’s correction for multiple comparisons. Data are presented as mean ± SEM.

CD38 is a multifunctional ectoenzyme with NAD^+^ glycohydrolase activity, converting NAD^+^ into ADP-ribose (ADPR) and nicotinamide. It also exhibits ADP-ribosyl cyclase and cyclic ADPR (cADPR) hydrolase activities, thereby influencing intracellular NAD^+^ availability and cellular metabolism^20,34^ (Figure 7F). Given that Rab4A promotes CD38 expression, we investigated whether Rab4A overexpression alters NAD^+^ metabolism through CD38.

To assess the impact of Rab4A and CD38 on NAD^+^ levels, we used targeted liquid chromatography tandem mass spectrometry (LC-MS/MS) to measure intracellular NAD^+^ concentrations in control, Rab4A^+^, and Rab4A^DN^ Jurkat cells. NAD^+^ levels were significantly reduced in Rab4A^+^ cells exhibiting elevated CD38 expression. However, CRISPR-mediated deletion of CD38 restored NAD^+^ levels in Rab4A^+^ CD38^KO^ Jurkat cells, indicating that CD38 is responsible for Rab4A-induced NAD^+^ depletion (Figure 7A). We also measured NADH concentrations, which were correspondingly reduced in Rab4A^+^ cells compared to controls. However, deletion of CD38 failed to restore NADH levels in Rab4A^+^ CD38^KO^ cells (Figure 7B), suggesting that the Rab4A-CD38 axis directly regulates NAD^+^ hydrolysis while independently affecting NADH levels via redox signaling^21^. Along these lines, Rab4A^+^ cells exhibited diminished NADP^+^ (**Supplementary Figure S7A**) and NADPH concentrations (**Supplementary Figure S7B**), which were also unresponsive to CD38 deletion (**Supplementary Figures S7A and S7B**). This suggests that Rab4A modulates NADPH independently of CD38. To further characterize Rab4A-CD38-mediated NAD^+^ metabolism, we performed untargeted LC-MS/MS and observed the depletion of cyclic ADP ribose (cADPR) (Figure 7C) and the accumulation of ADPR (Figure 7D) and nicotinamide (Figure 7E) in Rab4A^+^ Jurkat cells, all of which were all reversed by deletion of CD38 (Figures 7C–E). *In vivo*, serum from B6.TC/Rab4^Q72L^ mice showed elevated nicotinamide compared to controls, which was significantly reduced by treatment with the Rab inhibitor 3-(3-pyridyl)-2-hydroxy-2-phosphono propanoic acid (3-PEHPC)^9^ (**Supplementary Figure S8**), supporting a physiological role for Rab4A in modulating NAD^+^ metabolism *in vivo*. Additionally, Rab4A increased kynurenine levels (**Supplementary Figure S7C**), possibly reflecting enhanced flux through the tryptophan-NAD^+^ biosynthetic pathway as a compensatory response to NAD^+^ depletion. However, the simultaneous depletion of NADPH may impair the conversion of kynurenine to 3-hydroxykynurenine (**Supplementary Figure S7D**), disrupting downstream steps of the kynurenine pathway. These findings further support a role for CD38 in Rab4A-driven alterations in pyridine nucleotide metabolism. Furthermore, Rab4A promoted the accumulation of lactate and 2-hydroxyglutarate (**Supplementary Figures S9A and S9B**). CD38^KO^ reversed the accumulation of lactate but not 2-hydroxyglutarate (**Supplementary Figure S9A**), suggesting that enhanced glycolysis and lactic acid fermentation are CD38-dependent compensatory responses to NAD^+^ consumption, while the buildup of 2-hydroxyglutarate may stem from CD38-independent alterations in the TCA cycle. Together, these findings support a model in which Rab4A reprograms cellular metabolism through both CD38-dependent and CD38-independent mechanisms.

### Rab4A promote CD38-dependent STAT3 activation and FOXO1 expression

**Figure 8 |:**
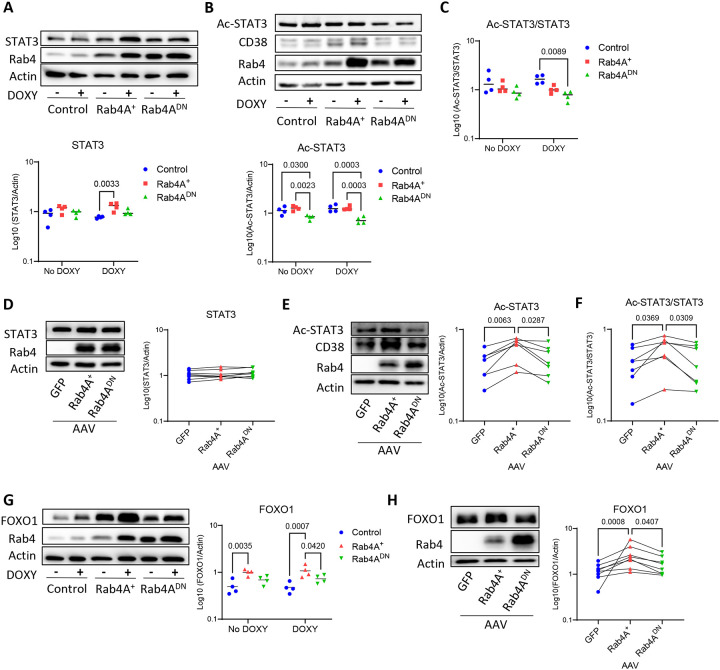
Rab4A promotes STAT3 activation and FOXO1 expression in Jurkat and primary human CD4^+^ T cells. Western blot analyses show that **(A-C, G)** in Jurkat cells (n = 4 independent cell culture replicates), **(A)** total STAT3 levels are increased in Rab4A^+^ cells compared to controls, **(B)** ac-STAT3 is reduced in Rab4A^DN^ relative to both control and Rab4A^+^ cells, and **(C)** the ac-STAT3/STAT3 ratio is significantly lower in Rab4A^DN^ compared to control and Rab4A^+^ cells. **(D-F, H)** In AAV-infected primary human CD4^+^ T cells from healthy donors (n = 8), **(D)** total STAT3 levels are unchanged in Rab4A^+^ cells, while **(E)** ac-STAT3 is elevated in Rab4A^+^ cells. **(F)** The ac-STAT3/STAT3 ratio is significantly higher in Rab4A^+^ cells than in controls. **(G-H)** FOXO1 expression is elevated in Rab4A^+^ cells compared to control and Rab4A^DN^ cells in both **(G**) Jurkat and **(H**) primary human CD4^+^ T cells from healthy donors. Statistical analyses were performed using two-way ANOVA with Tukey’s correction for multiple comparisons for **(A-C, G)** and paired one-way ANOVA with Tukey’s correction for multiple comparisons for **(D-F, H)**. Data are presented as mean ± SEM.

Since intracellular NAD^+^ availability regulates the activity of histone deacetylases such as Sirtuin-1^35^—which in turn modulates STAT3 acetylation and activity^36^—we next examined whether Rab4A contributes to the regulation of STAT3 activation, a key checkpoint of T cell activation and cytokine signaling^37^. Western blot analysis revealed that Rab4A increased total STAT3 protein levels in Jurkat cells, compared to controls (Figure 8A). In contrast, Rab4A^DN^ cells showed reduced levels of acetylated STAT3 (ac-STAT3) (Figure 8B). Quantification of the ac-STAT3/STAT3 ratio confirmed a significant decrease in Rab4A^DN^ Jurkat cells compared to controls (Figure 8C). In primary human CD4^+^ T cells transduced with AAV-Rab4A constructs, Rab4A did not significantly impact total STAT3 levels (Figure 8D). However, primary human Rab4A^+^ CD4^+^ T cells also exhibited significantly higher ac-STAT3 levels (Figure 8E) and ac-STAT3/STAT3 ratio (Figure 8F), suggesting reduced Sirtuin-1 activity in Rab4A^+^ cells, consistent with CD38-mediated NAD^+^ depletion. Since STAT3 binds to the promoter of the transcription factor, Forkhead Box O1 (FOXO1), which in turn suppresses IL-2 production in T cells^38^, we examined whether Rab4A and CD38 modulate FOXO1 expression. Indeed, Rab4A markedly increased FOXO1 protein levels both in Jurkat cells (Figure 8G) and primary human CD4^+^ T cells (Figure 8H) relative to controls.

**Figure 9 |:**
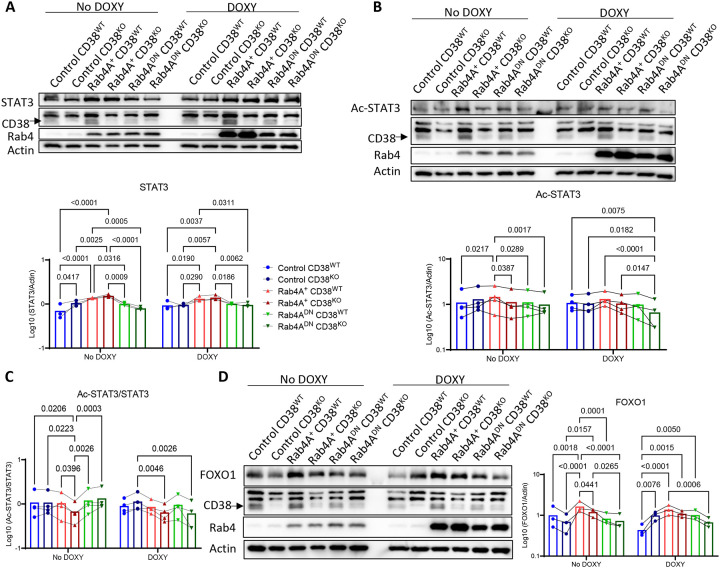
Rab4A promotes STAT3 activation and FOXO1 expression via CD38. Western blot analyses in Rab4A^+^ Jurkat cells show that CD38 knockout (CD38^KO^) compared to empty vector control (CD38^WT^), **(A)** does not alter STAT3 levels, **(B)** reduces acetylated STAT3 (ac-STAT3), and **(C)** decreases ac-STAT3/STAT3 ratio. **(D)** CD38^KO^ also reduces FOXO1 levels in Rab4A^+^ cells compared to CD38^WT^. Statistical analyses were performed using two-way ANOVA with Tukey’s correction for multiple comparisons. Data are presented as mean ± SEM.

To determine whether these effects were mediated by CD38, we assessed STAT3 and FOXO1 expression in Rab4A-modified Jurkat cells with CRISPR-mediated CD38 deletion (CD38^KO^). While CD38 deletion did not alter total STAT3 levels (Figure 9A), it significantly reduced ac-STAT3 (Figure 9B) and ac-STAT3/STAT3 ratio (Figure 9C). CD38 deletion also reversed Rab4A-induced FOXO1 activation (Figure 9D), supporting a role for CD38-driven FOXO1 expression in mediating IL-2 depletion by Rab4A. Together, these findings demonstrate that Rab4A promotes STAT3 activation and FOXO1 expression through CD38-dependent NAD^+^ metabolism. Given the critical roles of STAT3 and FOXO1 in cytokine production and T cell signaling, this pathway may underlie Rab4A-driven T cell dysfunction in SLE.

## DISCUSSION

In this study, we identify Rab4A as a potent regulator of CD38 expression and its downstream effects on mTOR activation, NAD^+^ metabolism, STAT3 activation, FOXO1 expression, and IL-2 suppression in CD4^+^ T cells. These findings provide mechanistic insight into how Rab4A contributes to the metabolic control of T cell development and lupus pathogenesis^3,9,10,21^.

We demonstrate that Rab4A promotes CD38 expression in both Jurkat and primary CD4^+^ T cells by enhancing its recycling and appearance in the plasma membrane. Reduced colocalization of CD38 with lysosomes suggests that Rab4A prevents CD38 degradation, and potentiates its recycling to the cell membrane, thus prolonging its signaling lifespan. Retention of CD38 expression via endocytic recycling is consistent with prior studies showing that CD38 internalization serves as a negative regulatory mechanism^25^. Since CD38 expression is upregulated in SLE T cells and correlates with disease activity^14,15^, our results implicate Rab4A as a potential driver of CD38 dysregulation in lupus.

Importantly, we show that CD38 is required for Rab4A-mediated mTOR activation. Rab4A facilitates the colocalization of CD38 with mTOR, as well as increased phosphorylation of mTORC1 and mTORC2 substrates, which are attenuated by CD38 deletion. Notably, CD38^+^ CD4^+^ T cells from SLE patients show elevated p4E-BP1 and pAKT1 levels relative to CD38^−^ CD4^+^ T cells, reinforcing the relevance of this axis in the context of SLE. These results support a model in which Rab4A promotes mTOR activation through CD38, contributing to aberrant mTOR signaling in T cells of SLE patients^3,10,39^ and lupus-prone mice^9,21^.

IL-2 is essential for maintaining immune tolerance by promoting T_reg_ differentiation^6^ and suppressing T_H_17 expansion^7^, and diminished IL-2 levels correlate with disease activity in SLE patients^40^. We also demonstrate that Rab4A suppresses IL-2 production via a CD38-dependent but mTOR-independent mechanism. While mTOR inhibition with rapamycin further reduced IL-2 and further increased CD38 expression, CD38 deletion alone restored IL-2 secretion by Rab4A-overexpressing cells. These findings point to the involvement of distinct mechanisms by which Rab4A-directed CD38 expression is capable of enhancing mTOR activation and IL-2 production.

Metabolomic analysis shows that Rab4A promotes CD38-mediated consumption of NAD^+^ and accumulation of its degradation products (nicotinamide, ADPR, cADPR), consistent with its known enzymatic functions^20^. *In vivo*, constitutively active Rab4A also elevated nicotinamide in lupus-prone mice, which was reversed by pharmacological Rab inhibition, independently linking endosome traffic to NAD^+^ metabolism. Moreover, Rab4A-induced increases in lactate and kynurenine suggest compensatory upregulation of glycolysis and tryptophan-dependent NAD^+^ biosynthesis pathways, further supporting a model of immunometabolic reprogramming via endosome traffic.

Mechanistically, we found that since NAD^+^ is a critical cofactor for the deacetylase Sirtuin-1^35^, as Rab4A-driven NAD^+^ depletion increased STAT3 acetylation^36^. Rab4A enhanced acetylated STAT3 levels, which were reversible by CD38 deletion, supporting a model in which Rab4A enhances STAT3 activity through NAD^+^ metabolism. Furthermore, Rab4A stimulates FOXO1 expression, which is also blocked by CD38 deletion. STAT3 drives proinflammatory T cell responses^37^ and promotes FOXO1 expression that suppresses IL-2 production^38^. Thus, these results link Rab4A-driven CD38 expression and NAD^+^ depletion to STAT3/FOXO1-mediated suppression of IL-2 production in SLE T cells. Prior studies showed that rapamycin treatment reduces disease activity in SLE patients^41–44^, which involves the expansion of T_reg_ cells *in vivo*^44^ and *in vitro*^45^. Given that rapamycin blocked IL-2 production in this study, increased production of this cytokine may not underlie T_reg_ expansion in rapamycin-treated SLE patients^44–46^.

Our data support a model in which Rab4A overexpression in SLE T cells enhances CD38 recycling and surface retention, leading to mTOR activation via traffic to the lysosome, where mTOR gets activated by sensing amino acid sufficiency. Alternatively, Rab4A causes mTOR-independent but CD38-dependent NAD^+^ depletion, STAT3/FOXO1 activation, and IL-2 depletion. These multipronged signal transduction pathways may synergistically contribute to the proinflammatory T cell lineage development observed in SLE^47^. As also shown by this study, Rab4A promotes CD38 recycling and surface expression in CD19^+^ B cells that likely underlie B cell activation, expansion of plasma cells, and enhanced autoantibody production in lupus-prone B6.TC/Rab4A^Q72L^ mice^21^.

Given the strong association between CD38 expression and disease activity in SLE^14^, pharmacological targeting of the Rab4A-CD38 axis—either through modulation of Rab4A-mediated endosome traffic, antibody-mediated or enzymatic inhibition of CD38, or dietary restoration of NAD^+^ levels—could represent synergistic therapeutic interventions in patients with SLE.

## METHODS

These studies were conducted in compliance with all ethical regulations and study protocols approved by the Institutional Review Boards of the State University of New York Upstate Medical University.

### Jurkat cell lines

Doxycycline-inducible, GFP-expressing Jurkat cell lines—control, Rab4A-overexpression (Rab4A^+^), and dominant-negative mutant Rab4A^S27N^ (Rab4A^DN^)—were created as previously described^26^. Transfected cells were maintained in media containing 200 μg/mL neomycin (G418) and 100 μg/mL hygromycin. GFP expression was monitored by flow cytometry in all experiments, and Rab4 protein expression was confirmed by Western blot. As the vectors exhibited some leaky Rab4A expression in the absence of doxycycline, experimental figures include both +/− doxycycline culture conditions where relevant.

### Rapamycin treatment of Jurkat cells

Control, Rab4A^+^, and Rab4A^DN^ Jurkat cells were treated with 100 nM rapamycin (dissolved in DMSO) or 0.1% DMSO vehicle control, with and without 1 μg/mL doxycycline, and incubated at 37°C for 24 hours. To assess intracellular IL-2 expression, 4 hours before the end of the incubation, cells were treated with 50 ng/mL phorbol 12-myristate 13-acetate (PMA; Sigma, catalog # P1585), 1 μg/mL ionomycin (Sigma, catalog # I9657), and 10 μg/mL brefeldin A (Sigma, catalog # B6542–5MG) to inhibit Golgi function and promote cytokine accumulation.

After 24 hours, cells were harvested for Western blot and flow cytometry analyses.

### CRISPR-mediated deletion of CD38

CD38 was knocked out (CD38^KO^) in the Rab4A construct-transfected Jurkat cells using the pCas-Guide-EF1a-RFP CRISPR vector (Origene, catalog # GE100018; 5’-ATCCTCGTCGTGGTGCTCGCGG-3’). The CRISPR vector was introduced into the Rab4A-transfected Jurkat cells using the Neon Transfection System (Invitrogen) following the manufacturer’s protocol for Jurkat microporation. 72 h post-transfection, RFP expression, and CD38 knockout were confirmed by flow cytometry. The brightest 5% of RFP^+^ cells were sorted using flow cytometry and maintained in a medium containing 200 μg/mL G418 and 100 μg/mL hygromycin. Bulk-sorted aliquots of CD38^KO^ cells were used in all experiments, with RFP and/or CD38 expression verified in each experiment.

### Mouse models of SLE

Autoimmunity-resistant C57BL/6 (B6) and lupus-prone B6.Sle1.Sle2.Sle3 triple congenic (B6.TC) mice^48^ were obtained from Jackson Laboratory (Bar Harbor, ME). *Rab4A* is overexpressed in T cells of patients with SLE, as well as in T and B cells of multiple lupus-prone strains—including MRL/lpr, NZB/W F1, and B6.TC mice—prior to the onset of antinuclear antibodies (ANA) or clinical signs of disease. To investigate the role of Rab4A, we utilized a previously developed constitutively active *Rab4A* mouse model^21^. These constitutively active Rab4A^Q72L^ mice express a GTPase-deficient Rab4A mutant locked in its active, GTP-bound state.

To generate T cell-specific Rab4A knockout mice, floxed Rab4A^Q72L^ mice were crossed with CD4^Cre^ transgenic mice, resulting in deletion of Rab4A in CD4^+^ CD8^+^ double-positive T cells in the thymus^49^, effectively knocking out *Rab4A* in T cells of Rab4A^Q72L/CD4Cre^ mice. These mice are referred to as Rab4A^Q72L^_-_KO mice. Rab4A^Q72L^, Rab4A^Q72L^_-_KO, and CD4^Cre^ control mice were subsequently crossed with B6.TC mice to evaluate the impact of Rab4A modulation in lupus-prone backgrounds. The presence of the three lupus susceptibility loci (Sle1:chr1, ~76cM; Sle2:chr4, ~39cM; and Sle3:chr7, ~15cM) was confirmed in collaboration with Dr. Laurence Morel and monitored across generations, as previously described^48^.

All animal experiments were conducted in accordance with the NIH Guide for the Care and Use of Laboratory Animals and approved by the Institutional Animal Care and Use Committee (IACUC).

20-week-old female lupus-prone mice were treated with 3-(3-pyridyl)-2-hydroxy-2-phosphono propanoic acid (3-PEHPC), a nonspecific Rab geranylgeranyl transferase inhibitor known to inhibit multiple Rab isoforms^9^, as selective inhibitors for individual Rab GTPases are limited. Mice were randomly assigned to receive either PBS (vehicle control) or 150 μg/kg 3-PEHPC dissolved in PBS, administered via subcutaneous injection three times per week for 12 weeks. The numbers of mice in each genotype were as follows: B6.TC untreated (n = 15), B6.TC 3-PEHPC treated (n = 14), B6.TC/Rab4A^Q72L^ untreated (n = 13), B6.TC/Rab4A^Q72L^ 3-PEHPC treated, B6.TC/Rab4A^Q72L^_-_KO untreated (n = 7) and B6.TC/Rab4A^Q72L^_-_KO 3-PEHPC treated (n = 7). To monitor potential toxicity, mice were weighed weekly. Serum was collected from via submandibular bleeding after treatment, and metabolites were analyzed using liquid chromatography tandem mass spectrometry (LC-MS/MS).

### Transduction of Rab4A by adeno-associated virus (AAV)

Peripheral blood mononuclear cells (PBMCs) were collected from 18 healthy donors (16 females and 2 males), all of whom self-identified as White. The average age was 45.0 years, with a standard deviation of 13.7 years (**Supplementary Table S3**). In healthy donors’ CD4^+^ T cells isolated from PBMCs using Untouched Human CD4^+^ T cells Dynabeads Kit (ThermoFisher Scientific, catalog # 11346D; Figure 2A) and bulk PBMCs, Rab4A mutants were transduced by infection with adeno-associated virus (AAV) carrying Rab4A-mutant cDNAs upstream of the internal ribosomal entry site (IRES) of the pAAV-IRES-hrGFP vector (Stratagene/Agilent Technologies, catalog # 240071), as described previously^26^. To produce Rab4A-expressing adeno-associated virus (AAV), HEK293 cells were co-transfected with Rab4A-containing pAAV-IRES-hrGFP plasmid, along with pAAV-RC, and pHelper plasmids (Staratgene), which provide the necessary helper functions for virus production. Cells were infected at a 1:1 multiplicity of infection (MOI), and >99% of cells infected with pAAV-Rab4A-IRES-hrGFP (Rab4A^+^), pAAV-Rab4A^S27N^_-_IRES-hrGFP (Rab4A^DN^), or pAAV-IRES-hrGFP control (GFP) virus were GFP-positive (Figure 2B). Maximal GFP expression was observed 24 h post-infection by flow cytometry.

### Confocal immunofluorescence microscopy

AAV-infected primary human CD4^+^ T cells were either cultured *in vitro*, unstimulated, or stimulated with CD3/CD28. At 24 hours post-stimulation, cells were harvested and incubated at 37°C for 1 hour with 5 μM LysoTracker Red (LTR; ThermoFisher Scientific, catalog # L7528) in complete RPMI 1640 medium (cRPMI). After two washes with cRPMI, 100,000 cells were seeded onto poly-L-lysine-mimicking CC^2^ glass Nunc^™^ Lab-Tek^™^ II CC2^™^ Chamber Slide System (ThermoFisher Scientific). Cells were allowed to adhere for 30 minutes at room temperature, then fixed with 1% paraformaldehyde (Electron Microscopy Sciences), permeabilized using Permeabilization Buffer (eBioscience), and stained with fluorescently labeled antibodies against CD38 (Biolegend, catalog # 303526) and mTOR (Cell Signal Technology, catalog # 5048S) or CD4 (BD, catalog # 557707) for 40 minutes at room temperature. After staining, cells were washed twice with permeabilization buffer and once with phosphate-buffered saline (PBS). Coverslips were mounted using PBS, and images were acquired immediately on a Leica SP8 confocal microscope using a 63x oil-immersion objective with sequential scanning and a 0.3 μm z-step size. Fluorescent signals were captured using spectral detectors, and sequential scanning was used to record GFP (excitation: 488 nm, emission: 510 nm), CD38-BV421 (excitation: 405 nm, emission: 421 nm), LTR (excitation: 577 nm, emission: 590 nm), and mTOR-Alexa Fluor 647 (excitation: 650 nm, emission: 671 nm). For image analysis, brightfield images were first imported into Ilastik^50^ for pixel-based machine-learning classification of cells and backgrounds. The resulting segmentation maps were imported into Fiji/ImageJ to generate cell masks, which were used to make each cell for subsequent analysis. Fluorescence intensity measurements were done in Fiji/ImageJ and colocalization analyses were performed using the BIOP JACoP plugin^51^.

### Recycling assay

Single-cell suspensions were prepared by gently crushing freshly harvested mouse spleens through a 70 μm mesh filter (Corning, catalog # 431751) into a sterile Petri dish containing 10 mL of complete RPMI 1640 culture medium (cRPMI). The cRPMI contained 10% fetal bovine serum (FBS), 100 U/mL penicillin, 100 μg/mL streptomycin, 10 μg/mL amphotericin B, 2 mM L-glutamine, 1 mM sodium pyruvate, and 10 mM HEPES. Suspensions were washed once in 10 mL of cRPMI and stored on ice until all spleens were harvested to be processed together. Cells were centrifuged at 300 × g for 8 minutes at 4°C. The supernatant was removed, and red blood cells were lysed by resuspending the pellet in 5 mL of ACK lysis buffer (150 mM NH_4_Cl, 10 mM KHCO_3_, 0.1 mM EDTA, pH 7.4) for 5 minutes. The solution was then diluted with 5 mL of cRPMI and centrifuged again at 300 × g for 8 minutes at 4°C. CD4^+^ T, CD8^+^ T, double-negative (DN) T, B, and non-T non-B cells were isolated from freshly isolated mouse splenocytes using Dynabeads Kit (ThermoFisher Scientific). Cell subset isolations were confirmed by staining cells with fluorochrome-conjugated antibodies to CD3, CD4, CD8, and CD19. A portion of freshly stained cells was also labeled with an antibody against CD38 and kept at 4°C as a baseline reference for flow cytometry. All antibodies used are listed in **Supplementary Table S1**. Unstained cells were stimulated with 100 nM phorbol 12,13-dibutyrate (PDBu) (Millipore Sigma, catalog # P1269) at 37°C for 1 hour to induce receptor internalization^26^. PDBu was then removed by washing the cells three times in 1 mL of ice-cold PBS. To block *de novo* protein synthesis, cycloheximide (50 μg/mL; Sigma-Aldrich, catalog # O1810) was added, and cells were then incubated at 4°C for 10 minutes. An aliquot of the cells was kept on ice, while the remaining cells were incubated at 37°C for 30, 60, or 120 minutes to allow surface receptor recycling. At each time point, cells were stained for the CD38 expression at 4°C for 30 minutes and analyzed by flow cytometry on a BD LSR II.

### Cytokine assays

To quantify cytokines secreted by Jurkat cells, *in vitro* stimulation was performed. As previously reported, anti-CD3 and PMA stimulation is optimal for inducing cytokine production in Jurkat cells^52^. Cell culture plates were first coated with goat anti-mouse IgG (Jackson ImmunoResearch, catalog # 115-005-003) and incubated at 37°C for 2 hours. Plates were then washed twice with PBS and coated with anti-CD3 (OKT3) antibody, followed by a 1-hour incubation at 37°C. After two additional PBS washes, Jurkat cells were added at a concentration of 0.5 × 10^6^/mL in complete PRMI. Phorbol 12-myristate 13-acetate (PMA; Sigma Aldrich, catalog # P1585) was added at a final concentration of 50 ng/mL, and cells were cultured for 24 hours.

After stimulation, cell cultures were harvested into 1.5 mL Eppendorf tubes and centrifuged at 400 × g for 8 minutes at 4°C. The supernatant was carefully collected without disturbing the pellet and transferred to a new tube. Cytokine levels were measured using the BD Cytometric Bead Array (CBA) Human T_H_1/T_H_2/T_H_17 Cytokine Kit (BD, catalog # 560484), following the manufacturer’s instructions. Data were acquired using a BD LSR II flow cytometer and analyzed using the FlowJo CBA plugin.

### Cytokine measurement in mouse sera

Serum was collected from mice via submandibular bleeding, and cytokine concentrations were quantified using the MILLIPLEX^®^ Mouse Cytokine/Chemokine Magnetic Bead Panel (Millipore Sigma, catalog # MCYTMAG-70K-PX32), according to the manufacturer’s instructions. Assays were performed by the Molecular Analysis Core Facility at SUNY Upstate Medical University.

### Flow cytometry of human peripheral blood mononuclear cells (PBMCs)

Studies involving SLE patients were conducted as part of a clinical trial (NCT00775476). PMBCs were isolated from peripheral blood from SLE patients and healthy donors matched for ethnicity, sex, and age (±10 years) (**Supplementary Table S4**). Cells were cultured *in vitro* either without stimulation or co-stimulated with CD3/CD28 for 16 hours. For stimulation, cell culture plates were first coated with goat anti-mouse IgG (Jackson ImmunoResearch, catalog # 115-005-003) and incubated at 37°C for 2 hours. Plates were then washed twice with PBS and coated with anti-CD3 (OKT3) antibody, followed by a 1-hour incubation at 37°C. After two additional PBS washes, PBMCs were added at a concentration of 1 × 10^6^/mL in complete PRMI. Anti-CD28 antibody (BD, catalog # 555725) was added at 1 μL per mL of media, and cells were cultured for 16 hours prior to flow cytometry analysis. T cell subsets were identified by surface staining with antibodies against CD3, CD4, CD8, CD19, CD45RA, CD45RO, CD62L, and CCR7 to mark different immune cell lineages. For analysis of cytokine production and mTOR activity, cells were fixed with 1% paraformaldehyde (Electron Microscopy), permeabilized using Permeabilization Buffer (eBioscience), and stained with fluorescently labeled intracellular antibodies. A complete list of all antibodies and dilutions used is provided in **Supplemental Table S1**.

### Cell fractionation and plasma membrane isolation

5×10^7^ Jurkat cells were harvested from culture, and plasma membrane, nuclear, cytosolic, and organelle membrane fractions were isolated using the Minute^™^ Plasma Membrane Protein Isolation and Cell Fractionation Kit (Invent Biotechnologies, catalog # SM-005) was used according to the manufacturer’s instructions. Protein concentrations were measured using the Bradford assay with a protein standard (Sigma, catalog # P5619–1VL).

### Western blot analyses

For whole-cell lysates, cells were harvested from culture and pelleted at 400 × g for 5 minutes at 4°C. After removing the supernatant, cells were lysed by resuspending 1×10^6^ cells in 50 μL of sample buffer composed of: 1x Laemmli sample buffer (Bio-Rad, catalog # 161–0747), 1x cell lysis buffer (Cell Signaling Technology, catalog # 9803S), 1 mM phenylmethylsulfonyl fluoride (PMSF), 55 μM β-mercaptoethanol, 1x Halt Protease and Phosphatase Inhibitor Cocktail (ThermoFisher Scientific, catalog # 78445). For lysates from cell fractions, 30 μg of protein was mixed with 30 μL of the same sample buffer. Lysates were boiled at 95°C for 5 minutes, separated by 12% SDSpolyacrylamide gel electrophoresis (SDS-PAGE), and transferred to nitrocellulose membranes. Membranes were blocked in 5% (v/v) dry skim milk (Fisher Scientific, catalog # DF0032-17-3) in TBST or 5% bovine serum albumin (BSA; Fisher Scientific, catalog # 50-178-1634) in TBST for detection of phosphorylated proteins. A complete list of primary antibodies and horseradish peroxidase-conjugated secondary antibodies, including dilutions, is provided in **Supplemental Table S2**. Automated densitometry was used to quantify the relative levels of protein expression using the Azure 600 Imaging System and analyzed with AzureSpot Pro image analysis software (Azure Biosystems).

### Metabolome analysis by targeted LC-MS/MS

3×10^6^ cells for each Jurkat cell line were washed in 1 mL of PBS and pelleted at 500 × g for 5 minutes at 4°C. Cell pellets were washed again in 1 mL of PBS, pelleted at 500 × g for 5 minutes at 4°C, and then the supernatants were discarded. Each pellet was resuspended in 400 μL of 80% methanol (precooled at −80°C), vortexed thoroughly, incubated at −80°C for 15 minutes, and centrifuged at 13,000 × g for 30 minutes at 4°C. A 100 μL aliquot of the supernatant was transferred to autosampler vials with fused silica inserts for LC-MS/MS analysis. The sample extracts were injected twice. During the first injection, NAD^+^, NADH, NADP^+^, and NADPH were quantified using a targeted selected ion monitoring (t-SIM) method with multiplexing in negative ionization mode. For the second injection, the mass spectrometer operated in polarity switching mode to enable untargeted metabolomic profiling. The scan range was *m*/*z* 61 – *m*/*z* 915 and *m*/*z* 70 – *m*/*z* 920 in positive and negative mode, respectively. All other mass spectrometric and liquid chromatographic parameters were identical for both injections, as described below. Samples were analyzed using a Thermo Scientific Q Exactive Hybrid Quadrupole-Orbitrap mass spectrometer coupled to a Thermo Scientific Vanquish HPLC, as described previously^21^. Briefly, chromatographic separation was achieved on a Waters Xbridge BEH Amide column (2.1 × 100 mm, 3.5 μm particle size). The mobile phase consisted of (A) 10 mM ammonium acetate and 7.5 mM ammonium hydroxide in 97:3 (v/v) water:acetonitrile and (B) acetonitrile. The chromatographic gradient and flow rate were as follows: 0 min (85% B, 0.15 mL/min), 1.5 min (85% B, 0.15 mL/min), 5.5 min (35% B, 0.15 mL/min), 10.0 min (35% B, 0.15 mL/min), 10.5 min (35% B, 0.30 mL/min), 14.5 min (35% B, 0.30 mL/min), 15.0 (85% B, 0.15 mL/min), 25.0 min (85% B, 0.15 mL/min). The injection volume was 5 μL, and the column oven temperature was maintained at 30°C. The heated electrospray ionization (HESI) source voltage was 3600 V and 2500 V in positive and negative mode, respectively. The sheath, auxiliary, and sweep gases were set to 30, 10, and 0 (arbitrary units), respectively. The auxiliary gas heater temperature was 120°C, and the ion transfer capillary temperature was 320°C. The S-lens radio frequency (RF) level was set to 55 (arbitrary units). The Orbitrap resolving power was 70,000, the automatic gain control (AGC) target was set to 3×10^6^, and the maximum injection time was 200 ms. All peak integrations were performed in Thermo TraceFinder 4.1. The target compounds were quantified using external calibration. Calibration standards were measured in the range of 10 – 10,000 μg/L, and the linear range was used for quantitation (R2 > 0.989). Negative controls were prepared by performing mock sample extractions. 5-Thio-glucose was added to the extraction solvent (2.25 μM) and was used as an internal standard for quality control purposes. A list of compounds that were putatively identified based on the theoretical exact mass of their deprotonated/protonated ion forms is given in **Supplementary Table S5**.

## Supplementary Material

Supplementary Files

This is a list of supplementary files associated with this preprint. Click to download.

- CD38manuscriptSupplementaryMaterialswFigs.pdf
- CD38manuscriptsourcedata.xlsx
